# Demonstration of pollinator-mediated competition between two native *Impatiens* species, *Impatiens noli-tangere* and *I. textori* (Balsaminaceae)

**DOI:** 10.1002/ece3.1431

**Published:** 2015-02-25

**Authors:** Nanako Tokuda, Mitsuru Hattori, Kota Abe, Yoshinori Shinohara, Yusuke Nagano, Takao Itino

**Affiliations:** 1 Department of Biology, Faculty of Science, Shinshu UniversityNagano, Japan; 2 Institute of Mountain Science, Shinshu UniversityNagano, Japan

**Keywords:** Bumblebees, improper pollen transfer, plant–plant interaction, pollination, reproductive interference

## Abstract

Plant–plant interspecific competition via pollinators occurs when the flowering seasons of two or more plant species overlap and the pollinator fauna is shared. Negative sexual interactions between species (reproductive interference) through improper heterospecific pollen transfer have recently been reported between native and invasive species demonstrating pollination-driven competition. We focused on two native *Impatiens* species (*I. noli-tangere* and *I. textori*) found in Japan and examined whether pollinator-mediated plant competition occurs between them. We demonstrate that *I. noli-tangere* and *I. textori* share the same pollination niche (i.e., flowering season, pollinator fauna, and position of pollen on the pollinator's body). In addition, heterospecific pollen grains were deposited on most stigmas of both *I. noli-tangere* and *I. textori* flowers that were situated within 2 m of flowers of the other species resulting in depressed fruit set. Further, by hand-pollination experiments, we show that when as few as 10% of the pollen grains are heterospecific, fruit set is decreased to less than half in both species. These results show that intensive pollinator-mediated competition occurs between *I. noli-tangere* and *I. textori*. This study suggests that intensive pollinator-mediated competition occurs in the wild even when interacting species are both native and not invasive.

## Introduction

Animal-pollinated plants provide flower visitors with rewards such as nectar, and pollinating animals in turn facilitate plant reproduction by dispersing pollen to conspecific plants. When two or more species whose flowering seasons overlap and share pollinators, two kinds of plant–plant interaction may occur, facilitation and competition, via the shared pollinators (Mitchell et al. 2009). Although a number of studies have examined pollinator-mediated plant competition (Waser 1978; Fishman and Wyatt 1999; Mitchell et al. 2009; Takakura and Fujii 2010; Muchhala and Thomson 2012; Ye et al. 2014), studies of plants with specialist pollinators have long neglected it as a possible selective agent of such specialization (Waser et al. 1996; Johnson and Steiner 2000; Gómez and Zamora 2006; Sargent and Ackerly 2008). Recently, however, pollinator-mediated plant competition has been recognized as a force promoting specialization of angiosperms to different pollinators (e.g., Muchhala et al. 2010, 2014).

Pollinator-mediated plant competition can decrease plant fitness by reducing pollinator quantity or quality, or both (Mitchell et al. 2009). A reduction of pollinator quantity occurs when one plant species, by attracting the shared pollinator, causes the pollinator visitation frequency to the other species to decrease. A reduction of pollinator quality occurs through interspecific pollen transfer, when the pollen of one plant species is transferred to the flowers of another (i.e., to heterospecific flowers), and the heterospecific pollen deposited on the stigmas of another species may reduce female fitness in that species. In fact, it has been demonstrated both theoretically and empirically that pollinator-mediated plant competition can decrease the fitness of the interacting plant species (Waser 1978; Waser and Fugate 1986; Galen and Gregory 1989; Natalis and Wesselingh 2012). Thus, pollinator-mediated plant competition may potentially influence the distribution and abundance of interacting plant species and erase the trace of competition. Therefore, we can predict that it is difficult to find ongoing competition between native species. In fact, many studies have focused largely on competition between native and invasive species, or the evolutionary consequences of past competition among native species (Yang et al. 2007; Eaton et al. 2012; Gibson et al. 2012; Muchhala and Thomson 2012 etc.). Therefore, ecological studies of pollinator-mediated plant competition among coexisting native species are important to understand their distribution and mechanisms of species coexistence.

In this study, we focused on two native *Impatiens* species (*I. noli-tangere* and *I. textori*) and quantified the occurrence of pollinator-mediated competition between the two species. These species are both native to Japan, and they often occur together and flower at the same time of the year in western Japan (M. Hattori pers. obs.), where both are pollinated by the bumblebee *Bombus diversus* (Kato 1988). We investigated the flowering season, pollinators, and the effect of improper pollen transfer in these two *Impatiens* species. Here, we address two questions: (1) Do *I. noli-tangere* and *I. textori* share their pollination niche (i.e., flowering season, pollinators, and sites of pollen deposition on the pollinator's body)? and (2) Is female fitness of both species decreased by interspecific competition (Does deposition of heterospecific pollen on stigmas reduce fruit set in both species)?

## Materials and Methods

### Study materials

**Figure 1:**
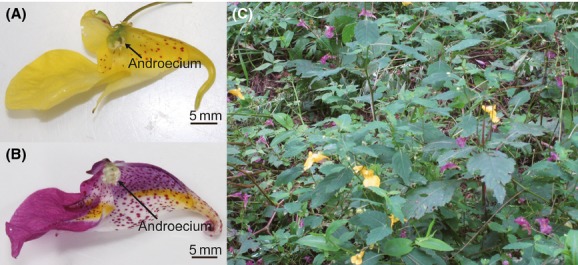
Flowers of (A) *Impatiens noli-tangere* and (B) *I. textori*. A part of the tubular corolla has been dissected away to show the androecium. (C) A mixed stand of the two *Impatiens* species at study site 2.

*Impatiens noli-tangere* L. and *I. textori* Miq. (Balsaminaceae) are annual herbs with very similar vegetative and floral morphology (Fig.1A and B). *Impatiens noli-tangere* is distributed across Europe, East Asia, and North America in moist habitats (e.g., along streams), and it has long-spurred yellow flowers (Fig.1A and C). *Impatiens textori* is distributed in Japan, the Korean Peninsula, and northeastern China in moist to wet habitats (e.g., along streams and in marshes), and it has long-spurred magenta flowers (Fig.1B and C).

Both *I. noli-tangere* and *I. textori* are visited primarily by *Bombus diversus* in western Japan (Kato 1988), and the anthers and stigmas of both species are located in the same position in the flower: The androecium is fused into a single unit that completely encloses the pistil (Satake 1999; Fig.1A and B).

A major difference in reproductive mode between *I. noli-tangere* and *I. textori* is the frequency of cleistogamous (CL) flowers. *Impatiens noli-tangere* produces not only chasmogamous (CH) flowers but also CL flowers, and the frequency of CL flowers depends on abiotic environmental factors such as light conditions (Masuda and Yahara 1994). In contrast, *I. textori* rarely produces CL flowers (Sugita 2001; Masuda et al. 2004). Another difference is that CH flowers in *I. noli-tangere* are protandrous, so they must be cross-pollinated, whereas CH flowers in *I. textori* are homogamous, so they must not be cross-pollinated (Sugita 2001; Iwaizumi and Sakai 2004; Masuda et al. 2004).

Although both *I. noli-tangere* and *I. textori* have the same number of chromosomes (2*n* = 20), hybrids between these species have never been reported, nor did we observe any hybrid-like individuals in our study area where *I. noli-tangere* and *I. textroi* co-occur.

We selected two study sites where both species occur: study site 1 (area, 38 m × 7 m; 36.2646°N, 137.8339°E; 1120 m a.s.l.) and study site 2 (area, 7.5 m × 1 m; 36.2827°N, 137.8299°E; 775 m a.s.l.). Both sites are approximately 2.0 km apart and are located at the edge of a *Cryptomeria japonica* forest in Nagano, central Japan.

### Flowering season, pollinators, and interspecific pollen transfer in the wild

#### Flowering season

At study site 1, we investigated whether the flowering seasons of *I. noli-tangere* and *I. textori* overlapped. For each species, we haphazardly selected and marked 40 plant individuals and checked whether they had flowers almost daily from 9 September to 5 October 2009.

At site 2, we investigated whether the flowering seasons of *I. noli-tangere* and *I. textori* overlapped by haphazardly selecting and marking plant individuals (*I. noli-tangere*: *n *=* *18, *I. textori*: *n *=* *7) and checking whether they had flowers almost weekly from 2 July to 7 October 2013.

#### Pollinators

To confirm that *I. noli-tangere* and *I. textori* shared pollinators at study site 1, on 6 days in late September 2009 when both species were in full bloom, we recorded the insect species that visited the flowers and touched the anthers for a 15-min period every hour from sunrise to sunset.

Furthermore, to confirm whether pollen grains of both species became attached to the same part of the bumblebee body, we randomly collected bumblebees visiting *Impatiens* flowers, anesthetized them using CO_2_, and then brushed away the pollen grains on their bodies. Then, after the bumblebees recovered from the anesthesia, we let them visit one flower, collected them again, and examined where on their bodies the pollen grains were attached. We examined eight bees for each *Impatiens* species.

#### Pollinator behavior

To observe pollinator behavior at site 2, on two fine days in early September 2008 when both plant species were in full bloom, we recorded flower visitation behavior of insects from 06:00 to 15:00 Japan Standard Time within a quadrate (1.7 m × 1 m) where 15 flowers on at least 10 individuals.

#### Pollen transfer between *I. noli-tangere* and *I. textori* in the wild

To confirm heterospecific pollen deposition on the stigmas of *I. noli-tangere* and *I. textori*, at study site 1, we haphazardly collected 25 flowers from plants of each species that were located no more than 2 m away from plants of the other species. We first cut the stigmas off the flowers and then sealed the stigmas with transparent nail enamel on glass slides (Matsumura and Washitani 2002). In the laboratory, we examined the stigmas under a stereoscopic microscope (SMZ1500, Nikon, Tokyo) and determined whether heterospecific pollen grains had been deposited on them. We identified the pollen of each species using explicit criteria based on pollen grain shape and size (Nakamura 1980).

### Experiments on reproductive interference

#### Greenhouse

To detect whether reproductive success was decreased by heterospecific pollen deposition on stigmas, we conducted hand-pollination experiments with *I. noli-tangere* in 2010 and with *I. textori* in 2008.

To eliminate the effect of self-fertilization, we emasculated the flowers of 20 haphazardly selected individuals of *I. noli-tangere* at study site 1. We collected pollen grains of each plant species and used a black swab to apply them to receptive stigmas of fully opened flowers: flowers received only conspecific pollen grains (*n *=* *17), only heterospecific pollen grains (*n *=* *10), or mixed pollen grains (*I. noli-tangere* pollen: *I. textori* pollen, w:w, 9:1, *n *=* *20; 5:5, *n *=* *20; and 1:9, *n *=* *21 (on 20 individuals)). After hand pollination, we measured fruit set as the percentage of flowers that produced fruit when all fruits had matured.

At study site 2, we randomly collected 14 *I. textori* seedlings and reared them in a greenhouse at Shinshu University, Matsumoto, Japan (650 m a.s.l.) until they flowered. We emasculated the flowers of 130 haphazardly selected flowers. We applied pollen grains of each plant species to receptive stigmas of fully opened flowers: flowers received only conspecific pollen grains (*n *=* *30), only heterospecific pollen grains (*n *=* *34), or mixed pollen grains (*I. noli-tangere* pollen:*I. textori* pollen, w:w, 1:9, *n *=* *22; 5:5, *n *=* *23; or 9:1, *n *=* *21 (on 14 individuals)). After hand pollination, we measured fruit set as the percentage of flowers that produced fruit when all fruits had matured.

In these experiments, we collected pollen grains from more than 10 individuals located without neighboring the other species within 2 m at study site 1 and 2. Collected pollen grains were taken to our laboratory and preserved in the refrigerator for 3 days. Because used pollen grains had high ability for fertility in the only conspecific pollen-pollinated treatment (see Results), the pollen grains maintained quality in these experiments.

#### In the wild

At study site 1, we haphazardly selected and marked 26 *I. noli-tangere* flowers that were located no more than 2 m away from flowering *I. textori,* and 30 *I. noli-tangere* flowers located without neighboring *I. textori* within 2 m. Similarly, we haphazardly selected 30 and 14 *I. textori* with and without neighboring *I. noli-tangere* flowers within 2 m, respectively. We measured fruit set of the flowers when the fruits matured. For each treatment, the flowers were selected from at least ten different individuals.

### Statistical analysis

We used JMP 9.0 software (SAS Institute, Inc., Cary, NC) for all statistical analyses. We used a logistic regression analysis to test the effects of heterospecific pollen grains on fruit set in each species. In this analysis, we used arcsine square root-transformed proportional data to improve normality and homoscedasticity (Sokal and Rohlf 1995). We evaluated the significance, defined as *P *<* *0.05, of the logistic regression analysis by calculating Wald chi-square values. We used chi-square tests to test the effect of neighboring heterospecific flowers on fruit set in the wild.

## Results

### Flowering season and pollinators

**Figure 2:**
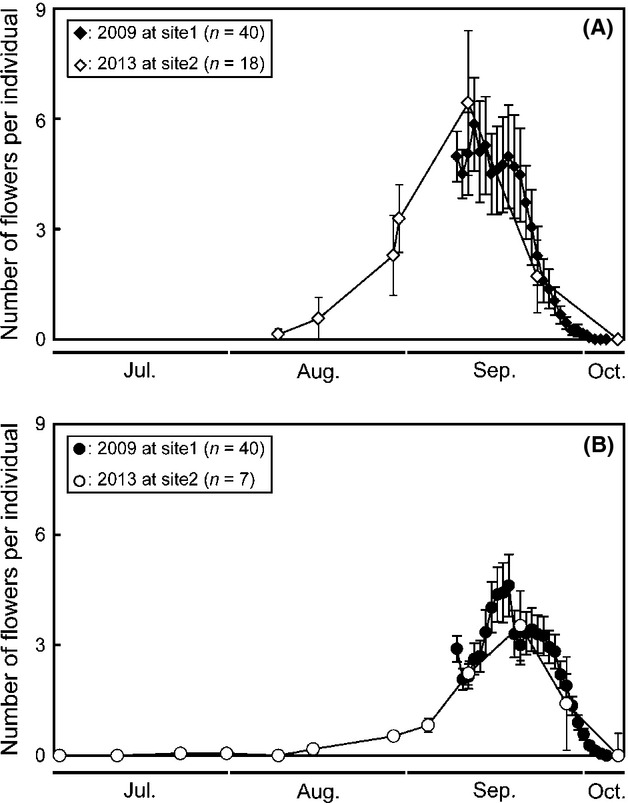
Seasonal change of average number of open flowers per individual (±standard error) in (A) *Impatiens noli-tangere* and (B) *I. textori* at study sites 1 and 2 in Japan.

*Impatiens noli-tangere* started to flower in late July and flowering finished in early October (Fig.2). *Impatiens textori* started to flower in early August and flowering finished in early October. Therefore, the flowering season of the two species virtually overlapped (Fig.2).

The major pollinator of both species is *B. diversus*, as Kato (1988) reported in Kyoto, western Japan. Pollen grains of both *Impatiens* species attached to the same parts of the bumblebee's body: the dorsal sides of the head, thorax, and upper abdomen.

### Pollen transfer between *I. noli-tangere* and *I. textori* in the wild

We found heterospecific pollen grains on most of the stigmas of both *I. noli-tangere* and *I. textori* (number of flowers having heterospecific pollen on the stigma/number of observed flowers: *I. noli-tangere*, 16/25; *I. textori*, 19/25).

### Pollinator behavior

We observed the behavior of 54 *B. diversus* individuals with 143 flower visitations (number of visitations = 55 and 88 to *I. noli-tangere* and *I. textori*, respectively) with 89 transition movements between flowers within the observation quadrate (the mean number of transition movement per bee was 1.65 ± 0.28 (standard error)). Among the 89 transition movements of *B. diversus* individuals, 68 were between conspecific flowers and 21 were between heterospecific flowers.

### Reduction of fruit set due to heterospecific pollen transfer in a greenhouse

**Figure 3:**
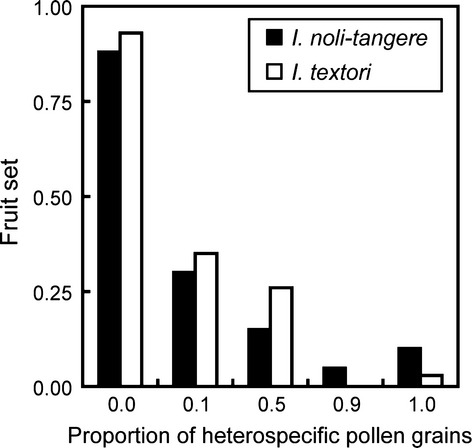
Fruit set in *Impatiens noli-tangere* and *I. textori* following hand pollination with conspecific pollen only, heterospecific pollen only, or mixtures of conspecific and heterospecific pollen. The abscissa shows the proportion of heterospecific pollen by weight in the pollen used for hand pollination.

Hand pollination with only conspecific pollen grains resulted in a high rate of fruit set in both species (number of fruits/number of hand-pollinated flowers: *I. noli-tangere*, 15/17; *I. textori*, 28/30; Fig.3). In contrast, hand pollination with a pollen mixture having as little as 10% heterospecific pollen grains greatly decreased fruit set (number of fruits/number of hand-pollinated flowers: *I. noli-tangere*, 6/20; *I. textori*, 7/20; Fig.3). Furthermore, fruit set was negatively correlated with the proportion of heterospecific pollen grains (Fig.3: *I. noli-tangere*, Wald *x*^2^ = 34.4; df = 1; *P *<* *0.001; *I. textori*, Wald *x*^2^ = 41.8; df = 1; *P *<* *0.001). Therefore, female fitness of both species was decreased by deposition of even a small proportion of heterospecific pollen grains on stigmas.

### Reduction of fruit set in relation to heterospecific neighborhood in the wild

In both species, fruit set within a heterospecific neighborhood was less than that of a pure stand neighborhood (*I. noli-tangere*: fruit set with heterospecific neighborhood = 5/26, fruit set without heterospecific neighborhood = 29/30, *x*^2^ = 47.60, *P *<* *0.001, *I. textori*: fruit set with heterospecific neighborhood = 7/30, fruit set without heterospecific neighborhood = 13/14, *x*^2^ = 21.50, *P *<* *0.001).

## Discussion

Our observations demonstrate that *I. noli-tangere* and *I. textori* share important pollination niche features (i.e., flowering season, pollinator, and pollen deposition sites on the pollinator's body) (Figs1 and 2). Furthermore, pollinators often transited between heterospecific flowers. Moreover, in both species, we observed that fruit set within a heterospecific neighborhood in the wild was less than that in a pure stand, and further, heterospecific pollen grains greatly decreased female fitness of both species in our hand-pollination experiment (Fig.3). These results indicate that intensive pollinator-mediated plant competition occurs between the native *Impatiens* species in the wild. Some studies have focused on competition between native species and invasive species (Nishida et al. 2012, 2014). In this study, we showed competition between native species. Further study is needed to focus on competition between native species, because competition between native species likely occurs much more frequently than expected.

The mechanism of decreased female fitness in the two native *Impatiens* species is not apparent. We suggest that three mechanisms of reproductive interference might cause the observed reduction in fruit set: (1) Heterospecific pollen limits the space on the stigma surface available for conspecific pollen (i.e., physical interference), (2) Heterospecific pollen allelopathically inhibits fertilization with conspecific pollen (i.e., physiological interference) (Morales and Traveset 2008; Mitchell et al. 2009), and (3) Ovules fertilized by heterospecific pollen are aborted (Arceo-Gómez and Ashman 2011; Runquist 2012). Our results suggest that the mechanism of interspecific competition is the latter two possibilities, because fruit set is dramatically decreased even when only 10% of the applied pollen grains are heterospecific (Fig.3). Further studies are needed to examine the mechanism of reproductive interference in these species. In particular, conspecific pollen tube growth speed in the style should be compared between pistils with and without heterospecific pollen grains on the stigma.

The pollination niche overlap between *I. noli-tangere* and *I. textori* is puzzling because reproductive competition sometimes theoretically excludes such spatiotemporal niche overlaps (Waser 1978; Groning and Hochkirch 2008; Muchhala et al. 2010; Takakura and Fujii 2010). We suggest two possible explanations for the coexistence of these competing *Impatiens* species: (1) Either or both species may have traits that reduce negative effect of reproductive interference and/or (2) other mortality factors are stronger than pollinator-mediated plant competition in delimiting the distribution of the two species (e.g., seed predation).

With regard to hypothesis (1), reproductive character displacement (e.g., divergence of flowering season, divergence of the floral morph, and self-fertilization) can potentially reduce the influence of reproductive interference (Waser 1978; Armbruster et al. 1994; Fishman and Wyatt 1999; Muchhala et al. 2010). In these two *Impatiens* species, the influence of reproductive interference might be reduced by self-pollination of CH flowers in *I. textori* (Sugita 2001; Masuda et al. 2004; Yuan et al. 2004) or by the production of CL flowers in *I. noli-tangere* (Sugita 2001; Masuda et al. 2004). These traits may mitigate the influence of competition between the two species. To further examine hypothesis (1), comparison of the investment in CL flowers by *I. noli-tangere* between areas in which both *Impatiens* species are found and those in which only *I. noli-tangere* occurs is essential.

Although many studies focus on pollinator-mediated plant competition between native and invasive species (e.g., Brown et al. 2002; Gibson et al. 2012; Nishida et al. 2012), there has been few studies focusing on strictly native species. This may be because pollinator-mediated plant competitions are more easily observed between invasive species and native species than that between two native species. As pollinator-mediated plant competitions potentially influence the distribution and trait evolution of interacting plant species, they may erase the trace of competition between two native species that have long coexisted through selection to reduce reproductive interference. Some segregating mechanisms are expected to evolve if competition reduces reproductive success. In fact, some studies have revealed the mechanism to avoid pollinator-mediated plant competition (Fishman and Wyatt 1999; Aizen and Vázquez 2006; Hopkins et al. 2012; Huang and Shi 2013). For example, Hopkins et al. (2012) proposed evidences that divergence of flower color was caused by natural selection for reducing maladaptive hybridization in *Phlox* species. Furthermore, Huang and Shi (2013) found that pollen placement on the pollinator's body was different between three *Pedicularis* species sharing the same pollinator niche. As such segregation mechanisms likely reduce the negative effect of pollinator-mediated plant competition, they obscure evidence of past competition. Therefore, further study is needed to reveal how pollinator-mediated plant competition has evolutionary and ecological significance in the interaction system including *I. noli-tangere, I. textori*, and the pollinating bee, *Bombus diversus*.
